# Design and Evaluation of Niacin Microspheres

**DOI:** 10.4103/0250-474X.59549

**Published:** 2009

**Authors:** Vidyavathi Maravajhala, Nirmala Dasari, Asha Sepuri, S. Joginapalli

**Affiliations:** Institute of Pharmaceutical Technology, Sri Padmavati Mahila Visvavidyalayam (Women's University), Tirupati-517502, India; 1 Natco Pharma Ltd, Banjara Hills, Hyderabad-500 033, India

**Keywords:** Encapsulation, ethyl cellulose, *in vitro*, microspheres, niacin, w/o/o double emulsion

## Abstract

Present study aims to prepare and evaluate niacin microspheres. Niacin-ethyl cellulose microspheres were prepared by water-in-oil-in-oil double emulsion solvent diffusion method. Spherical, free flowing microspheres having an entrapment efficiency of 72% were obtained. The effect of polymer-drug ratio, surfactant concentration for secondary emulsion process and stirring speed of emulsification process were evaluated with respect to entrapment efficiency, *in vitro* drug release behavior and particle size. FT-IR and DSC analyses confirmed the absence of drug-polymer interaction. The *in vitro* release profile could be altered significantly by changing various processing and formulation parameters to give a controlled release of drug from the microspheres. The percentage yield was 85%, particle size range was 405 to 560 μm. The drug release was controlled for 10 h. The *in vitro* release profiles from optimized formulations were applied on various kinetic models. The best fit with the highest correlation coefficient was observed in Higuchi model, indicating diffusion controlled principle. The *in vitro* release profiles of optimized formulation was studied and compared with commercially available niacin extended release formulation.

Microspheres can be defined as solid approximately spherical and polymeric particles ranging in size from 1 to 100 μm. They are made from polymeric, waxy or other protective materials such as natural, semisynthetic and synthetic polymers and are used as drug carrier matrices for drug delivery. Niacin, (pyridine-3-carboxylic acid) is a highly water soluble drug. Niacin in large doses, blocks the breakdown of fats in adipose tissue, thus altering blood lipid levels, so it is used in the treatment of hyperlipidemia. The half life of the drug is 20-45 min. Usual oral dosage regimen is 500 to 2000 mg in 2 to 4 divided doses in a day with maximum dosage of 2000 mg/day. To achieve maximum therapeutic effect with a low risk of adverse effects, controlled preparations are preferred[1]. The side effects could be lowered by controlling the drug release and by adjusting the absorption rate. This can be achieved by employing suitable modifications in the manufacturing process[2,3]. Thus to reduce the frequency of administration, to reduce incidence of facial flush and to improve patient compliance, a controlled release formulation of niacin is required. So far a few extended release matrix tablets of niacin were prepared and available in the market[4], but no other approaches like microspheres, nanoparticles which provide more advantages than matrix tablets are attempted with niacin. Microspheres provide more uniform distribution with reduced dose dumping compare to matrix tablets, so, present work aimed at design and evaluation of niacin microspheres. Ethyl cellulose is a semi synthetic polymer which is a non-biodegradable and biocompatible polymer and is an extensively studied encapsulating material for the controlled release of pharmaceuticals. Several researchers have investigated the utilization of ethyl cellulose as a polymer to microencapsulate a drug by coacervation phase separation technique[5–8], emulsion solvent evaporation technique[9].

The use of w/o/o double emulsion solvent diffusion method to microencapsulate the drugs were reported[9]. The present study was aimed at design and evaluation of microspheres of highly water soluble niacin by water-in-oil-in-oil (w/o/o) double emulsion solvent diffusion method using ethyl cellulose as the retardant material with high entrapment efficiency and prolonged release. Various processes and formulation parameters such as drug-polymer ratio, stirring speed and surfactant concentration were optimized to maximize the entrapment and to prolong the drug release. These microspheres were evaluated for drug content and *in vitro* drug release. Drug-polymer interactions in the solid state were studied by Fourier Transform infra red spectroscopy (FTIR), differential scanning calorimetry and UV spectroscopy. The surface characteristics were evaluated by scanning electron microscopy (SEM).

## MATERIALS AND METHODS

Niacin was received as a gift sample from Natco Pharma Ltd., Hyderabad, India. Ethyl cellulose (14 cps viscosity grade), dichloromethane, acetonitrile, light paraffin and pet ether were also used in the study. All the reagents and solvents used were of analytical grade and were obtained from S.D. Fine Chem. Ltd., Mumbai, India, satisfying pharmacopoeial standards.

### Preparation of microspheres by double emulsion solvent diffusion method:

All microspheres were prepared according to the formulae given in Table 1 by w/o/o double emulsion solvent diffusion method. Weighed amounts of ethyl cellulose and niacin were dissolved in 30 ml of a mixture of acetonitrile and dichloromethane (1:1). The initial w/o emulsion was formed by adding 2 ml of deionized water to the drug-polymer solution by constant stirring at 500 rpm for 5 min. The w/o primary emulsion was then slowly added to light liquid paraffin containing span 80 as a surfactant with constant stirring for 2 h. The pet ether (10 ml) was added to harden the formed microspheres and stirring was further continued for 1 h. The resulting microspheres were separated by filtration, made it free from liquid paraffin by washing with pet ether (50 ml) and finally air dried over a period of 12 h[10].

**TABLE 1:** EFFECT OF VARIOUS PARAMETERS ON MEAN PARTICLE SIZE AND ENTRAPMENT EFFICIENCY

Formulation code	Formulation parameter	Theoretical drug content %	Experimental drug content %	Entrapment efficiency %	Mean particle size μm
	Ethyl cellulose: Niacin ratio				
C1	1:0.2	17.6	6.68	37±1.7	548
C2	1:0.3	23	16.17	72±1.3	405
C3	1:0.5	33.3	26.6	79±0.8	498
C4	1:0.75	42.86	37.26	86±0.5	527
C5	1:1	50	32.43	64.9±0.7	560
	Surfactant concentration (% w/v)				
C2	0.5	23	16.17	72±0.9	405
C6	1.0	23	13.69	59±0.8	524
C7	2.0	23	11.95	51±1.8	282
	Stirring speed of secondary emulsification (rpm)					
C2	500	23	16.17	72±1.67	405
C8	1000	23	19.65	85±0.90	281
C9	1500	23	12.86	56±0.8	202

C1 to C5 formulations vary in polymer to drug ratio, C2, C6 and C7 formulations vary in surfactant concentrations prepared with constant polymer to drug ratio (1:0.3) and C2 C8 and C9 formulations differ in stirring speeds of emulsifications with same formulae.

Different ethylcellulose:niacin ratios (1:0.25, 1:0.5, 1:0.75, 1:1, 1.5:1, 2:1, 3:1 and 3.5:1) were used in order to investigate the effect of polymer:drug ratio on release and the entrapment efficiency of microspheres. Various concentrations of span 80 (0.5%, 1% and 2%) and various stirring speeds (500, 1000 and 1500 rpm) were used to investigate the influence of surfactant concentration and effect of stirring rate on release and entrapment efficiency of microspheres.

### Size, shape and surface analysis[11]

Size distribution of the prepared microspheres was studied by optical microscopic technique. SEM photographs were taken using Scanning electron microscope model (SEM), Hitachi 5520, at 10 kv to study the shape and surface morphology. The photographs were observed for morphological characteristics and to confirm spherical nature of the microspheres.

### Micromeritic properties

Tap density of the prepared microspheres was determined using tap density tester and % Carr's index was calculated. Angle of repose was assessed to know the flowability of microspheres by a fixed funnel method[12].

### Entrapment efficiency[10]

An accurately weighed quantity of microspheres were crushed into powder and added to 100 ml of distilled water. Mixture was kept for 92 h. Then the solution was filtered and drug content was estimated by UV spectrophotometer at 262 nm. The drug entrapment efficiency was determined by using the formula, drug entrapment efficiency = (experimental drug content/theoretical drug content)×100….(1). The statistical difference in entrapment efficiency of prepared formulations was determined by student's t-test. The percentage yield of microsphere was also calculated based on the quantities of polymer, drug used in microspheres.

### Interaction studies:

Drug-polymer interactions were studied by UV spectroscopy, IR spectroscopy and DSC analysis. The UV spectra of pure niacin solution in distilled water and solution prepared for determination of entrapment efficiency of microspheres were recorded in the range of 220 to 320 nm using systronic 106 UV/Vis spectrophotometer. The FTIR spectra were recorded for pure niacin, blank microspheres and drug loaded microspheres using thermo elution FTIR spectrophotometer. The scanning range was 400 to 4000 cm^-1^. The DSC analysis of pure niacin, blank microspheres and niacin loaded microspheres was carried out in the heating range of 40° to 300° at a rate of 10° min^-1^ using TAG 1000 Differential scanning calorimeter.

### *In vitro* release studies:

The USP paddle type dissolution test apparatus was used for all *in vitro* release studies of all prepared formulations. A weighed quantity of microspheres was placed in 900 ml of distilled water taken in a dissolution vessel. The dissolution medium was stirred at 50 rpm and maintained at constant temperature (37±1°). At predetermined time intervals, 5 ml samples were withdrawn and concentration of drug was estimated using UV spectrophotometer at 262 nm. An equal volume of fresh dissolution medium was replaced after withdrawal of each sample. Percentage drug dissolved and dissolution rate of selected prepared microspheres (C2, C8) was compared with commercial niacin extended release tablets Niaspan (Abbott laboratories, North Chicago, IL, USA). All the release studies were carried out in triplicate.

### Release kinetics[12]:

In order to investigate the mechanism of niacin release from microsphere of ethyl cellulose:niacin (1:0.3) prepared at different stirring speeds (500 (C2) and 1000 (C8) rpm), the release data was analyzed with the following mathematical models, zero order (Eqn 1), first order (Eqn 2), Higuchi (Eqn 3) and Korsmeyer-Peppas (Eqn 4). Q_t_ = k_o_t (1), logQ_t_ = logQ-kt/2.303 (2), Q_t_ = kt^1/2^ (3) and M^t^/M^∞^ = kt^n^ (4), where Qt is the percent of drug released at time t, Q_o_ is the initial amount of drug present in the microspheres, M^t^/M^∞^ is the fraction of drug release, n is the release exponent and k is the constant of equations. Dissimilarity and similarity factors were calculated for comparison of dissolution profiles of prolonged release formulation (C_2_) and Niaspan with two eqns.

(2) $$f_{1}=\left\{\left[\sum_{t=1}\left|R_{t}-T_{t}\right|\right]/\left[\sum_{t=1}R_{t}\right]\right\}\times 100$$

(3) $$f_{2}=50\times \log\left\{{\left[1+\left(\frac{1}{n}\right)\sum {\left|R_{t}-T_{t}\right|}^{2}\right]}^{-0.5}\times 100\right\}$$

where n=number of dissolution time points, R_t_ = % drug dissolved from reference (Niaspan) formulation, T_t_ = % drug dissolved from test (C_2_) formulation

## RESULTS AND DISCUSSION

Niacin, a hydrophilic drug leads to low entrapment efficiency when encapsulated using aqueous phase as the processing medium[13]. Depending on the processing conditions, as much as 80% of the water soluble drug can partition out into the outer processing medium. The present study was aimed at encapsulation of niacin with sufficient high entrapment efficiency by w/o/o double emulsion solvent diffusion method using a non-aqueous processing medium. The primary requirement of this method to obtain microspheres is that the selected solvent system for polymer be immiscible with non-aqueous processing medium[14]. Acetonitrile is a unique organic solvent, which is a polar, water miscible and oil immiscible, so used for emulsion formation. All other polar solvents like methanol, ethanol, ethyl acetate, acetone and dimethylsulfoxide are oil immiscible and will not form emulsions of the polymer solution in oil. By using oil as the processing medium and acetonitrile alone as a solvent did not ensure formation of primary emulsion of the aqueous phase in the polymer solution. Immediately on mixing, the water miscibility of the acetonitrile brought about the precipitation of the polymer (ethyl cellulose). Hence, a non-polar solvent, namely dichloromethane was included with acetonitrile to decrease the polarity of the polymer solution.

In addition to this, it was also desirable that the second solvent be oil miscible, so that solvent removal is facilitated through extraction by processing medium leaving behind a viscous polymer solution. The optimal proportion of acetonitrile and dichloromethane was found to be 1:1 which enabled emulsion formation and yielded good microspheres[10]. No surfactant was used for stabilizing primary emulsion, since ethyl cellulose has the additional property of stabilizing w/o emulsion[15]. Span 80, a non-ionic surfactant was used to stabilize the secondary emulsification process. It has the HLB value of 4.3 and is expected to have a high disparity for the present emulsion system by reducing the surface tension at the interface.

Microspheres for formulations of C1, C2, C3, C4 and C5 were prepared with different polymer:drug concentrations of 1:0.2, 1:0.3, 1:0.5, 1:0.75 and 1:1, respectively. The increase in entrapment efficiency was achieved by increasing polymer-drug ratio from 1:0.25 to 1:0.75. With further increase in polymer-drug ratio from 1:0.75 to 1:1, significant (Table 1) decrease in encapsulation efficiency was observed (*P*<0.05, student t-test). The high drug loading typically results in lower encapsulation efficiency due to high concentration gradient resulting the drug to diffuse out of the polymer/solvent droplets to the external processing medium. The viscosity of the polymer solution at high drug loading was very high and was responsible for the formation of larger polymer/solvent droplets. It caused a decreased rate of entrapment of drug due to slower hardening of the larger particles, allowing time for drug diffusion out of the particles which tends to decrease the encapsulation efficiency[10]. At high polymer concentration (C1), entrapment efficiency is less may be due to dilution of the drug in polymer.

**Fig. 1:**
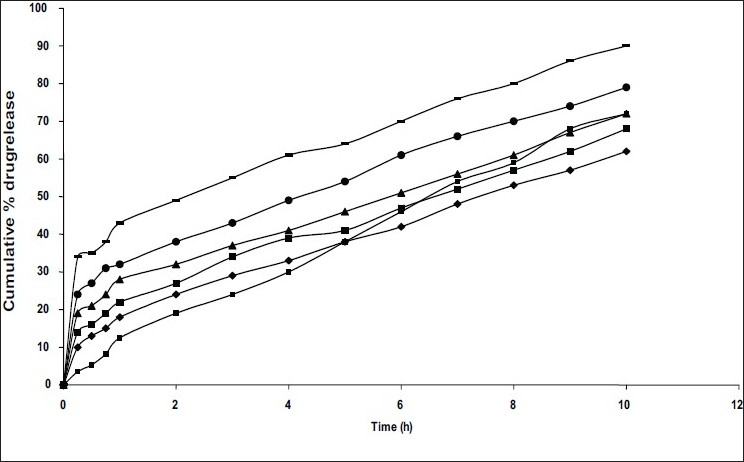
Comparative *in vitro* dissolution profiles of niacin from microspheres prepared with different polymer:drug ratios Microspheres with different polymer to drug ratios include (●) C1, 1:0.2; (▲) C2, 1:0.3; (■) C3,1:0.5; (◆) C4,1:0.75; (▬) C5,1:1 and (▪) Niaspan, a commercial niacin extended release tablet

These formulations exhibited initial burst effect in *in vitro* release which was due to the presence of drug particles on the surface of the microspheres. The initial burst effect may be attributed as a desired effect to ensure initial therapeutic plasma concentrations of drug. Drug release rate was increased with increasing amounts of niacin in the formulation. As more drug is released from the microspheres, more channels are probably produced, contributing to faster drug release rates. In addition, higher drug levels in the microsphere formulation produced a higher drug concentration gradient between the microspheres and dissolution medium, thus drug release rate was increased (fig. 1). *In vitro* studies also shown that drug release rate was decreased as increasing the polymer concentration due to its retarding effect for drug release.

Among the different polymer-drug ratios investigated, C2 formulation (1:0.3) is selected as it shown the optimum prolonged release (68% within 10 h and 100% within 18 h) with desired entrapment efficiency (72%). The similarity factor (f_2_) 63 was achieved with C2 formulation when compared to commercial extended release formulation. The mean particle size of different formulations differ in polymer concentration was in the range of 405 to 560 μm (Table 1).

**Fig. 2:**
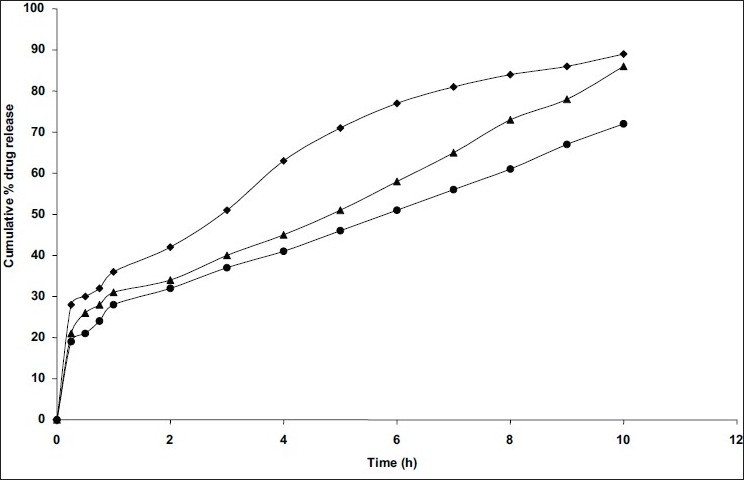
Comparative *in vitro* dissolution profiles of niacin from microspheres prepared with different concentrations of surfactant Microspheres prepared with 1:0.3 polymer:drug ratio and different concentrations of surfactant Span 80 include (●) C2, 0.5%; (▲) C6,1% and (◆) C7,2%

Microspheres for formulations of C6 and C7 were prepared by changing the surfactant (Span 80) concentration at 1% and 2%, respectively. Keeping the drug-polymer ratio constant, significant (p< 0.05, student's t-test) decrease in encapsulation efficiency of microspheres was observed with increasing the concentration of surfactant for secondary emulsification (Table 1). This may be due to the fact that the increase in surfactant concentration proportionately increases miscibility of acetonitrile with liquid paraffin (processing medium) which may increase the extraction of niacin into the processing medium. As the concentration of Span 80 increased, a faster drug release rate was observed (fig. 2). This may be attributed to the presence of more free drug on the surface of the microspheres with the increasing the concentration of span 80 used for secondary emulsification[10].

**Fig. 3:**
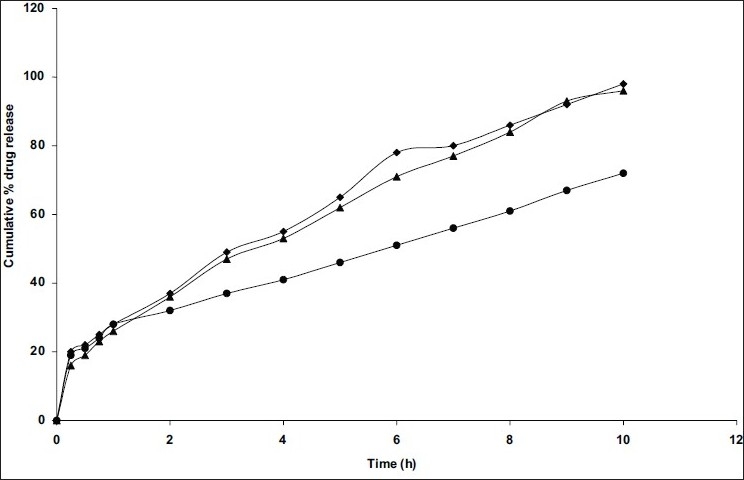
Comparative *in vitro* dissolution profiles of niacin from microspheres prepared at different stirring speeds Microspheres prepared with 1:0.3 polymer:drug ratio and 0.5% Span 80 at different stirring speeds of secondary emulsification include (●) C2, 500 rpm; (▲) C8, 1000 rpm; (◆) C9, 1500 rpm

**Fig. 4:**
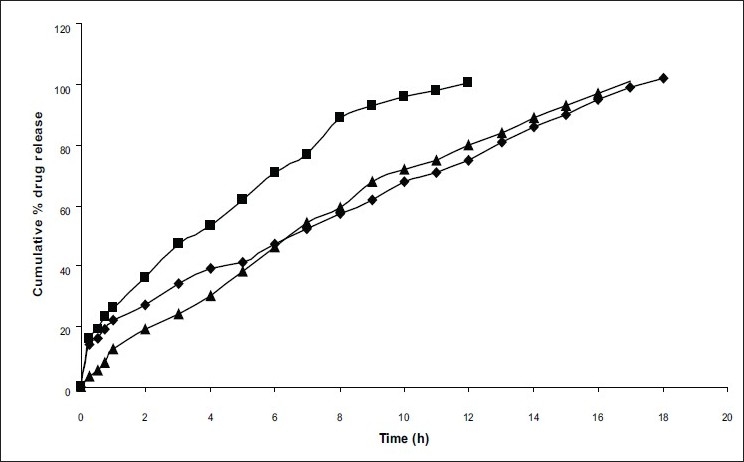
Comparative *in vitro* dissolution profiles of niacin from different selected prepared microspheres and commercial tablet. Prepared microspheres include, (◆) C2; (■) C8 and commercial tablet includes (▲) Niaspan

The mean particle size of formulations differ in surfactant concentration which was in the range of 282-524μm (Table 1). As concentration of surfactant was increased, the particle size was decreased which may be due to the formation of small globules during emulsification by span 80. The important factor that influences the size distribution of microspheres is the optimum stirring speed, thus the influence of stirring speed on C2 formulation at stirring speeds of 500, 1000 and 1500 rpm was studied by preparing microspheres (C2, C8 and C9) at different stirring speeds, respectively. The highest entrapment efficiency was observed with the stirring speed of 1000 rpm. The change of stirring speed from 1000 to 500 or to 1500 rpm, decreased the entrapment efficiency (Table 1) due to the formation of larger and smaller emulsion droplets respectively ensuring the drug diffusion out of the microspheres before they harden. Among these three formulations, C8 showed the highest entrapment efficiency. *In vitro* release studies revealed that drug release rate was increased with increasing the stirring speed may be due to decreased particle size, therefore surface area was increased (fig. 3). By comparing the dissolution rates of selected formulations (C2 and C8) with commercial extended release niacin tablets (niaspan), C2 formulation shown prolonged released as that of niaspan tablets (fig. 4) but had low entrapment efficiency as per Table 1 where as C8 formulation shown fast release rate than niaspan with high entrapment efficiency and small size. The mean particle size was decreased from 405 μm to 281 and 202 μm (Table 1) as speed is increased from 500 to 1000 and 1500 rpm, respectively this may be due to decreased globule size during emulsification.

Further, to confirm the mechanism of drug release, C2 and C8 formulations which have prolonged drug release and high entrapment efficiency with less mean particle size were subjected to release kinetic studies and its respective R^2^ values are given in Table 2. The best fit with the highest correlation coefficient was shown in Higuchi, Zero order equation as given in Table 2. The regression value is closer to unity in case of zero-order plot (R^2^ = 0.9846, 0.9574), so, the release is apparently zero order. The release of the drug followed zero-order release kinetics and regression value indicated fair linearity in the data. The data indicated poor linearity, and was less than the value of zero order plot, when it was plotted according to the first order equation.

**TABLE 2:** KINETIC PARAMETER (R^2^) OF SELECTED NIACIN MICROSPHERES

Plot	C2	C8
Zero order plot	0.984	0.957
First order plot	0.825	0.927
Higuchi plot	0.980	0.989
Korsmeyer Peppas plot	0.596	0.513

C2 and C8 formulations of same formula but operated at 500 and 1000 rpm, respectively

As high correlation was observed in the Higuchi plot, the drug release was proportional to square root of time, indicating that the drug release from ethyl cellulose microsphere was diffusion controlled. The data obtained were also put in Korsmeyer-Peppas model in order to find ‘n’ value, which describes the drug release mechanism. The ‘n’ value was 0.679 indicating drug release was zero order controlled by non-Fickian transport[11].

**Fig. 5:**
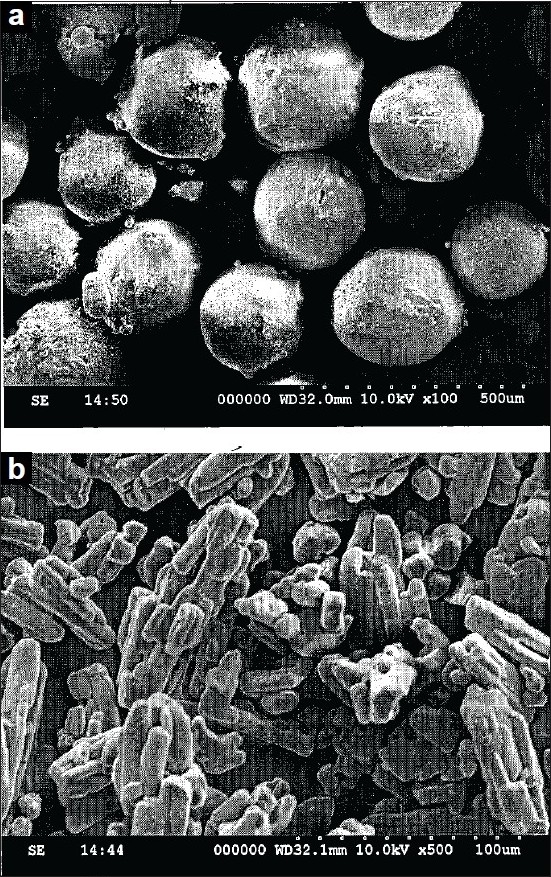
Scanning electron microscopic photographs SEM photographs showing surface morphology of a) niacin-loaded microspheres and b) pure niacin

The microspheres were found as spherical in shape and free flowing by surface topography investigation using SEM (fig. 5). Generally the microparticulate drug delivery systems are formulated as unit dosage forms in the form of capsule or tablet. So, such systems should possess better and adequate micromeritic properties.

The micromeritic properties and % yield of C2 and C8 formulations were estimated as these have optimum release rate and entrapment efficiency with desired size. Microspheres exhibited an angle of repose in the range of 26 to 28°θ which indicated good flow property (Table 3). The true density and bulk density values were in the range of 0.47 to 0.59 g/cm^3^ and 0.42 to 0.51 g/cm^3^, respectively. Using these values Carr's index was calculated, and the range was 10.6 to 13.5 indicated prepared microspheres possess excellent flow properties. The percentage yield was 85 to 92 (Table 3). Based on the results of micromeritic properties it can be concluded that the prepared microspheres have good flow properties.

**TABLE 3:** MICROMERITIC PROPERTIES OF SELECTED NIACIN MICROSPHERES

Formulation code	Angle of repose ±S.D	Bulk density ±S.D g/cm^3^	True density ±S.D g/cm^3^	Carr's Index ±S.D	% yield
C2	28±0.42	0.42±0.15	0.47±0.2	9.2±0.4	85
C8	26±0.23	0.51±0.10	0.59±0.3	13.5±0.8	92

C2 and C8 formulations of same formula but operated at 500 and 1000 rpm, respectively

The compatibility of niacin with ethyl cellulose in microspheres was evaluated by UV spectroscopy, FTIR and DSC analysis. The UV spectra of pure drug solution and the dissolution medium after drug release study were identical and the characteristic λmax of pure niacin was appeared at 262 nm on the UV spectra. It indicates that there were no drug-polymer interactions. This was further confirmed by FTIR and DSC analysis.

The study of FTIR spectra of niacin loaded ethyl cellulose microspheres demonstrated that the characteristic absorption peaks for carboxylic acid at 3434 cm^-1^, aromatic hydrogen at 2924 cm^-1^, carbonyl group at 1732 cm^-1^, imine group at 1366.8 cm^-1^ and C-H bending at 694 cm^-1^. The absorption peaks for microspheres were almost similar to those obtained for the pure drug.

**Fig. 6:**
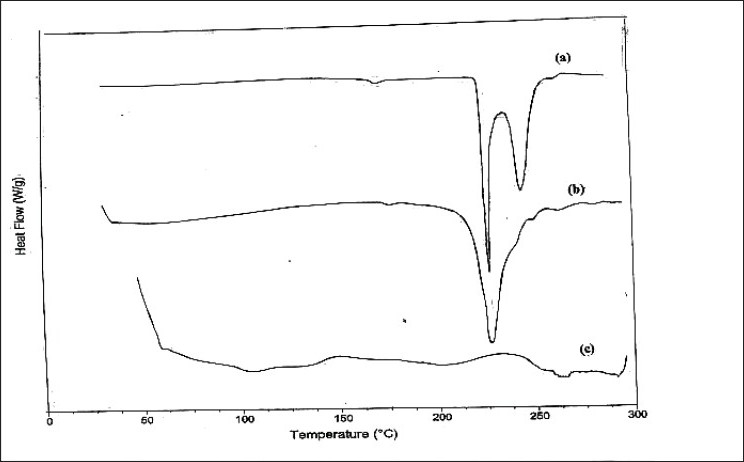
A differential scanning calorimetric scan DSC thermograms of (a) pure niacin, (b) niacin-loaded microspheres and (c) blank microspheres

The DSC curves of pure niacin, niacin-loaded ethyl cellulose microspheres and blank ethyl cellulose microspheres were shown in fig. 6. It was evident from the DSC profile that pure niacin exhibited a sharp endothermic peak at 236.6° which corresponds to the reported melting temperature of the drug (fig. 6). Another peak is also observed in DSC profile of pure drug may be due to its polymorphic form as the drug undergoes polymorphism. The DSC profile of niacin-loaded ethyl cellulose microspheres showed a peak at the temperature corresponding to niacin melting point but with the loss of its sharp appearance. It may be due to reduced drug crystallinity (fig. 6). There was no endothermic peak at that temperature in DSC profile of blank microspheres (fig. 6), which further confirmed the peak in microspheres is due to niacin.

The attempt to prepare controlled release microspheres of niacin with an optimum entrapment efficiency, size and prolonged release was successful and was comparable to commercially available niacin extended release formulation.
